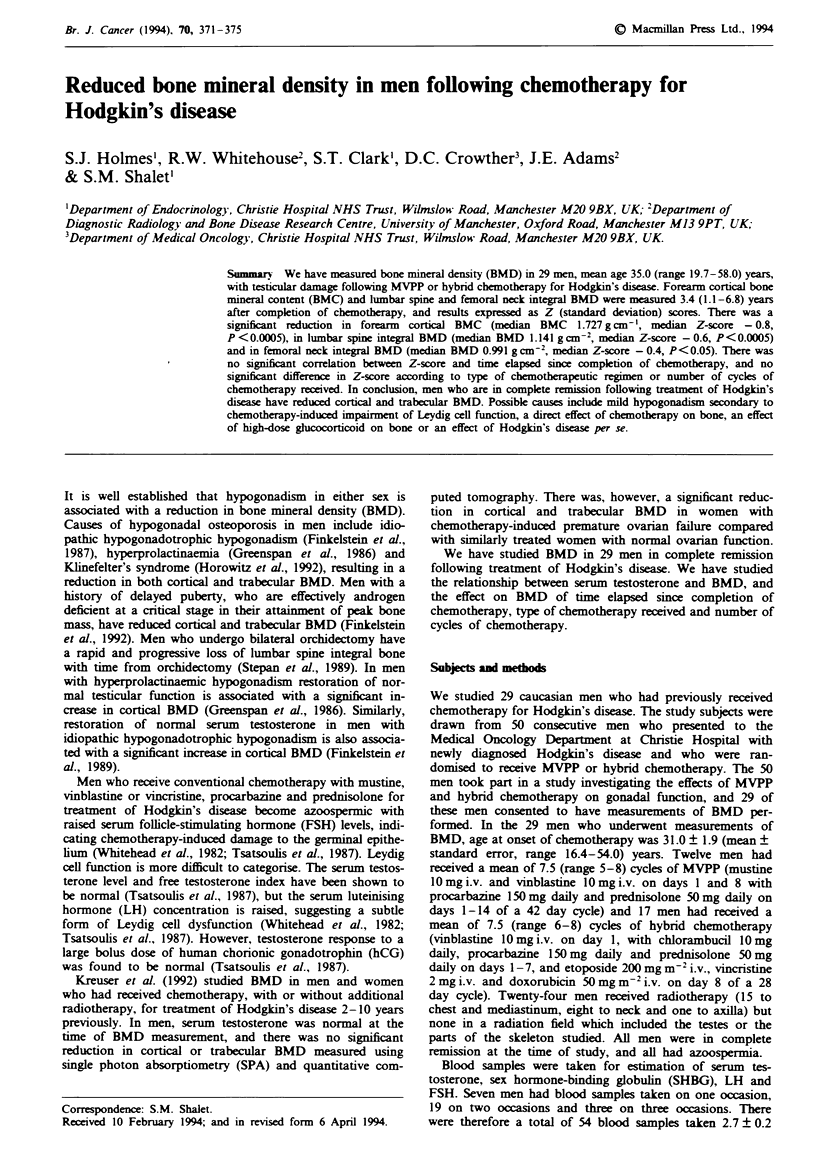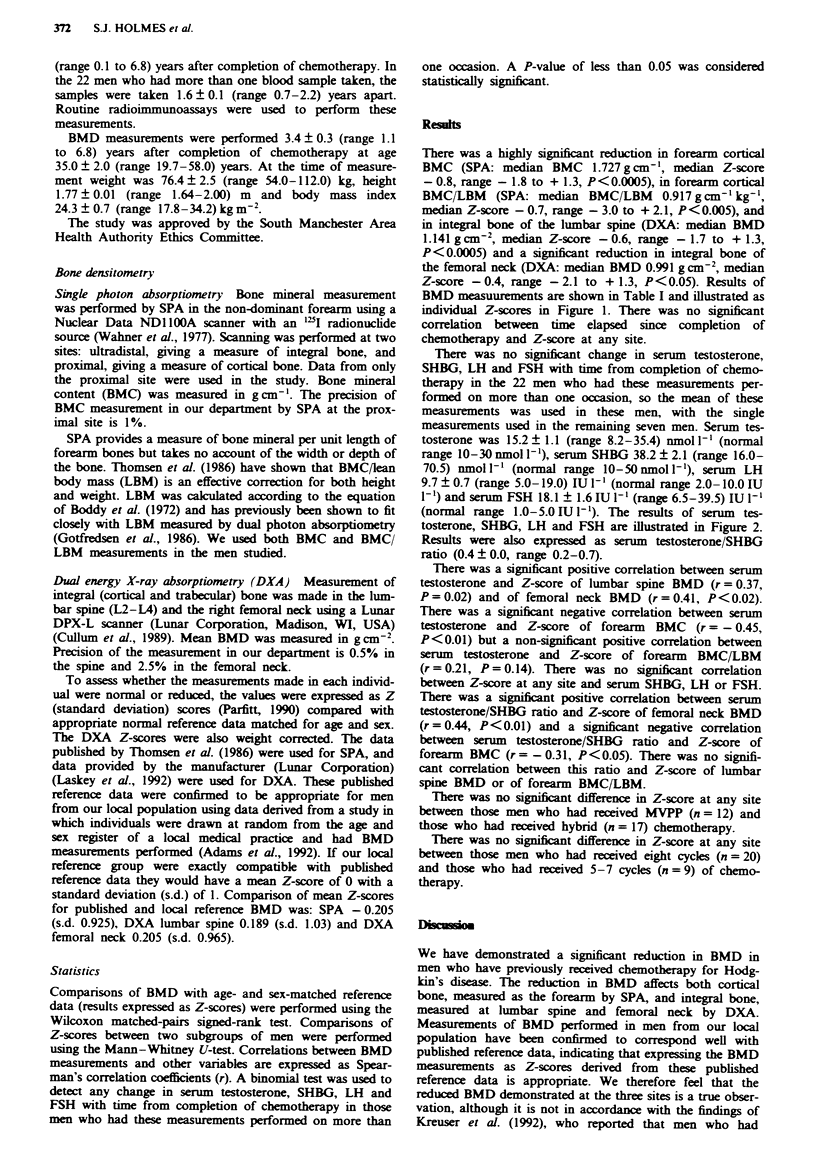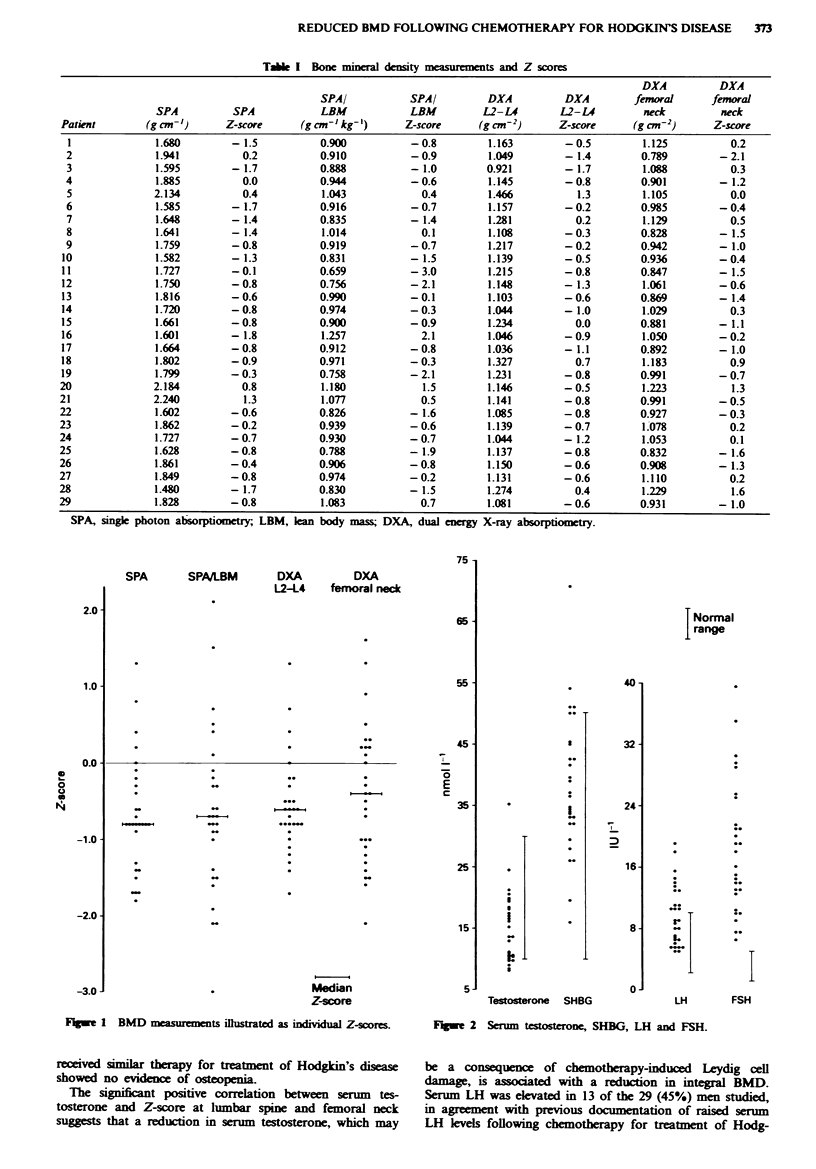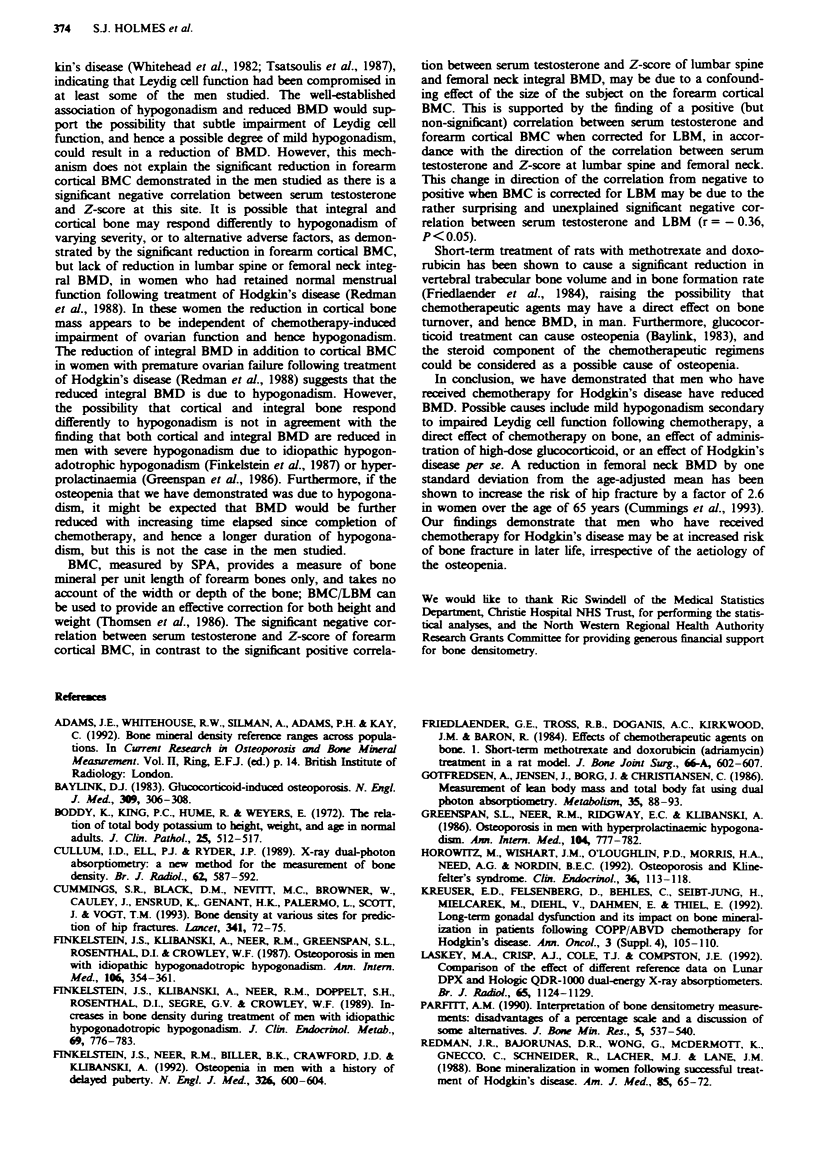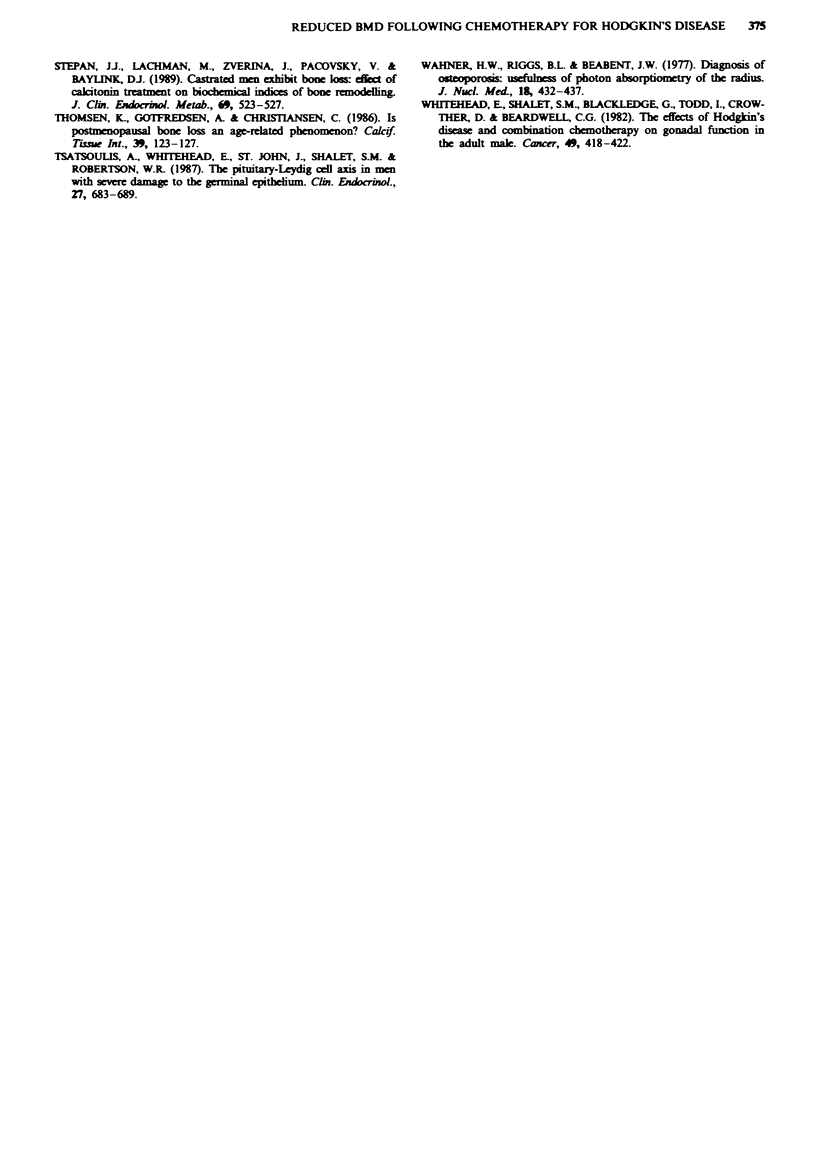# Reduced bone mineral density in men following chemotherapy for Hodgkin's disease.

**DOI:** 10.1038/bjc.1994.308

**Published:** 1994-08

**Authors:** S. J. Holmes, R. W. Whitehouse, S. T. Clark, D. C. Crowther, J. E. Adams, S. M. Shalet

**Affiliations:** Department of Endocrinology, Christie Hospital NHS Trust, Manchester, UK.

## Abstract

We have measured bone mineral density (BMD) in 29 men, mean age 35.0 (range 19.7-58.0) years, with testicular damage following MVPP or hybrid chemotherapy for Hodgkin's disease. Forearm cortical bone mineral content (BMC) and lumbar spine and femoral neck integral BMD were measured 3.4 (1.1-6.8) years after completion of chemotherapy, and results expressed as Z (standard deviation) scores. There was a significant reduction in forearm cortical BMC (median BMC 1.727 g cm-1, median Z-score -0.8, P < 0.0005), in lumbar spine integral BMD (median BMD 1.141 g cm-2, median Z-score -0.6, P < 0.0005) and in femoral neck integral BMD (median BMD 0.991 g cm-2, median Z-score -0.4, P < 0.05). There was no significant correlation between Z-score and time elapsed since completion of chemotherapy, and no significant difference in Z-score according to type of chemotherapeutic regimen or number of cycles of chemotherapy received. In conclusion, men who are in complete remission following treatment of Hodgkin's disease have reduced cortical and trabecular BMD. Possible causes include mild hypogonadism secondary to chemotherapy-induced impairment of Leydig cell function, a direct effect of chemotherapy on bone, an effect of high-dose glucocorticoid on bone or an effect of Hodgkin's disease per se.


Br. J. Cancer (1994), 70, 371–375 © Macmillan Press Ltd., 1994

### Reduced bone mineral density in men following chemotherapy for Hodgkin's disease

S.J. Holmes[1], R.W. Whitehouse[2], S.T. Clark[1], D.C. Crowther[3], J.E. Adams[2] & S.M. Shalet[1]

[1]*Department of Endocrinology, Christie Hospital NHS Trust, Wilmslow Road, Manchester M20 9BX, UK;* [2]*Department of Diagnostic Radiology and Bone Disease Research Centre, University of Manchester, Oxford Road, Manchester M13 9PT, UK;* [3]*Department of Medical Oncology, Christie Hospital NHS Trust, Wilmslow Road, Manchester M20 9BX, UK.*

**Summary** We have measured bone mineral density (BMD) in 29 men, mean age 35.0 (range 19.7–58.0) years, with testicular damage following MVPP or hybrid chemotherapy for Hodgkin's disease. Forearm cortical bone mineral content (BMC) and lumbar spine and femoral neck integral BMD were measured 3.4 (1.1–6.8) years after completion of chemotherapy, and results expressed as *Z* (standard deviation) scores. There was a significant reduction in forearm cortical BMC (median BMC 1.727 g $cm^{-1}$, median *Z*-score −0.8, $P<0.0005$), in lumbar spine integral BMD (median BMD 1.141 g $cm^{-2}$, median *Z*-score −0.6, $P<0.0005$) and in femoral neck integral BMD (median BMD 0.991 g $cm^{-2}$, median *Z*-score −0.4, $P<0.05$). There was no significant correlation between *Z*-score and time elapsed since completion of chemotherapy, and no significant difference in *Z*-score according to type of chemotherapeutic regimen or number of cycles of chemotherapy received. In conclusion, men who are in complete remission following treatment of Hodgkin's disease have reduced cortical and trabecular BMD. Possible causes include mild hypogonadism secondary to chemotherapy-induced impairment of Leydig cell function, a direct effect of chemotherapy on bone, an effect of high-dose glucocorticoid on bone or an effect of Hodgkin's disease *per se*.

It is well established that hypogonadism in either sex is associated with a reduction in bone mineral density (BMD). Causes of hypogonadal osteoporosis in men include idiopathic hypogonadotrophic hypogonadism (Finkelstein *et al.*, 1987), hyperprolactinaemia (Greenspan *et al.*, 1986) and Klinefelter's syndrome (Horowitz *et al.*, 1992), resulting in a reduction in both cortical and trabecular BMD. Men with a history of delayed puberty, who are effectively androgen deficient at a critical stage in their attainment of peak bone mass, have reduced cortical and trabecular BMD (Finkelstein *et al.*, 1992). Men who undergo bilateral orchidectomy have a rapid and progressive loss of lumbar spine integral bone with time from orchidectomy (Stepan *et al.*, 1989). In men with hyperprolactinaemic hypogonadism restoration of normal testicular function is associated with a significant increase in cortical BMD (Greenspan *et al.*, 1986). Similarly, restoration of normal serum testosterone in men with idiopathic hypogonadotrophic hypogonadism is also associated with a significant increase in cortical BMD (Finkelstein *et al.*, 1989).

Men who receive conventional chemotherapy with mustine, vinblastine or vincristine, procarbazine and prednisolone for treatment of Hodgkin's disease become azoospermic with raised serum follicle-stimulating hormone (FSH) levels, indicating chemotherapy-induced damage to the germinal epithelium (Whitehead *et al.*, 1982; Tsatsoulis *et al.*, 1987). Leydig cell function is more difficult to categorise. The serum testosterone level and free testosterone index have been shown to be normal (Tsatsoulis *et al.*, 1987), but the serum luteinising hormone (LH) concentration is raised, suggesting a subtle form of Leydig cell dysfunction (Whitehead *et al.*, 1982; Tsatsoulis *et al.*, 1987). However, testosterone response to a large bolus dose of human chorionic gonadotrophin (hCG) was found to be normal (Tsatsoulis *et al.*, 1987).

Kreuser *et al.* (1992) studied BMD in men and women who had received chemotherapy, with or without additional radiotherapy, for treatment of Hodgkin's disease 2–10 years previously. In men, serum testosterone was normal at the time of BMD measurement, and there was no significant reduction in cortical or trabecular BMD measured using single photon absorptiometry (SPA) and quantitative computed tomography. There was, however, a significant reduction in cortical and trabecular BMD in women with chemotherapy-induced premature ovarian failure compared with similarly treated women with normal ovarian function.

We have studied BMD in 29 men in complete remission following treatment of Hodgkin's disease. We have studied the relationship between serum testosterone and BMD, and the effect on BMD of time elapsed since completion of chemotherapy, type of chemotherapy received and number of cycles of chemotherapy.

### Subjects and methods

We studied 29 caucasian men who had previously received chemotherapy for Hodgkin's disease. The study subjects were drawn from 50 consecutive men who presented to the Medical Oncology Department at Christie Hospital with newly diagnosed Hodgkin's disease and who were randomised to receive MVPP or hybrid chemotherapy. The 50 men took part in a study investigating the effects of MVPP and hybrid chemotherapy on gonadal function, and 29 of these men consented to have measurements of BMD performed. In the 29 men who underwent measurements of BMD, age at onset of chemotherapy was 31.0 ± 1.9 (mean ± standard error, range 16.4–54.0) years. Twelve men had received a mean of 7.5 (range 5–8) cycles of MVPP (mustine 10 mg i.v. and vinblastine 10 mg i.v. on days 1 and 8 with procarbazine 150 mg daily and prednisolone 50 mg daily on days 1–14 of a 42 day cycle) and 17 men had received a mean of 7.5 (range 6–8) cycles of hybrid chemotherapy (vinblastine 10 mg i.v. on day 1, with chlorambucil 10 mg daily, procarbazine 150 mg daily and prednisolone 50 mg daily on days 1–7, and etoposide 200 mg $m^{-2}$ i.v., vincristine 2 mg i.v. and doxorubicin 50 mg $m^{-2}$ i.v. on day 8 of a 28 day cycle). Twenty-four men received radiotherapy (15 to chest and mediastinum, eight to neck and one to axilla) but none in a radiation field which included the testes or the parts of the skeleton studied. All men were in complete remission at the time of study, and all had azoospermia.

Blood samples were taken for estimation of serum testosterone, sex hormone-binding globulin (SHBG), LH and FSH. Seven men had blood samples taken on one occasion, 19 on two occasions and three on three occasions. There were therefore a total of 54 blood samples taken 2.7 ± 0.2

Correspondence: S.M. Shalet.
Received 10 February 1994; and in revised form 6 April 1994.

(range 0.1 to 6.8) years after completion of chemotherapy. In the 22 men who had more than one blood sample taken, the samples were taken 1.6 ± 0.1 (range 0.7–2.2) years apart. Routine radioimmunoassays were used to perform these measurements.

BMD measurements were performed 3.4 ± 0.3 (range 1.1 to 6.8) years after completion of chemotherapy at age 35.0 ± 2.0 (range 19.7–58.0) years. At the time of measurement weight was 76.4 ± 2.5 (range 54.0–112.0) kg, height 1.77 ± 0.01 (range 1.64–2.00) m and body mass index 24.3 ± 0.7 (range 17.8–34.2) kg $m^{-2}$.

The study was approved by the South Manchester Area Health Authority Ethics Committee.

*Bone densitometry*

*Single photon absorptiometry* Bone mineral measurement was performed by SPA in the non-dominant forearm using a Nuclear Data ND1100A scanner with an $^{125}I$ radionuclide source (Wahner *et al.*, 1977). Scanning was performed at two sites: ultradistal, giving a measure of integral bone, and proximal, giving a measure of cortical bone. Data from only the proximal site were used in the study. Bone mineral content (BMC) was measured in g $cm^{-1}$. The precision of BMC measurement in our department by SPA at the proximal site is 1%.

SPA provides a measure of bone mineral per unit length of forearm bones but takes no account of the width or depth of the bone. Thomsen *et al.* (1986) have shown that BMC/lean body mass (LBM) is an effective correction for both height and weight. LBM was calculated according to the equation of Boddy *et al.* (1972) and has previously been shown to fit closely with LBM measured by dual photon absorptiometry (Gotfredsen *et al.*, 1986). We used both BMC and BMC/LBM measurements in the men studied.

*Dual energy X-ray absorptiometry (DXA)* Measurement of integral (cortical and trabecular) bone was made in the lumbar spine (L2–L4) and the right femoral neck using a Lunar DPX-L scanner (Lunar Corporation, Madison, WI, USA) (Cullum *et al.*, 1989). Mean BMD was measured in g $cm^{-2}$. Precision of the measurement in our department is 0.5% in the spine and 2.5% in the femoral neck.

To assess whether the measurements made in each individual were normal or reduced, the values were expressed as *Z* (standard deviation) scores (Parfitt, 1990) compared with appropriate normal reference data matched for age and sex. The DXA *Z*-scores were also weight corrected. The data published by Thomsen *et al.* (1986) were used for SPA, and data provided by the manufacturer (Lunar Corporation) (Laskey *et al.*, 1992) were used for DXA. These published reference data were confirmed to be appropriate for men from our local population using data derived from a study in which individuals were drawn at random from the age and sex register of a local medical practice and had BMD measurements performed (Adams *et al.*, 1992). If our local reference group were exactly compatible with published reference data they would have a mean *Z*-score of 0 with a standard deviation (s.d.) of 1. Comparison of mean *Z*-scores for published and local reference BMD was: SPA − 0.205 (s.d. 0.925), DXA lumbar spine 0.189 (s.d. 1.03) and DXA femoral neck 0.205 (s.d. 0.965).

*Statistics*

Comparisons of BMD with age- and sex-matched reference data (results expressed as *Z*-scores) were performed using the Wilcoxon matched-pairs signed-rank test. Comparisons of *Z*-scores between two subgroups of men were performed using the Mann–Whitney *U*-test. Correlations between BMD measurements and other variables are expressed as Spearman's correlation coefficients ($r$). A binomial test was used to detect any change in serum testosterone, SHBG, LH and FSH with time from completion of chemotherapy in those men who had these measurements performed on more than one occasion. A *P*-value of less than 0.05 was considered statistically significant.

### Results

There was a highly significant reduction in forearm cortical BMC (SPA: median BMC 1.727 g $cm^{-1}$, median *Z*-score − 0.8, range − 1.8 to + 1.3, $P < 0.0005$), in forearm cortical BMC/LBM (SPA: median BMC/LBM 0.917 g $cm^{-1}$ $kg^{-1}$, median *Z*-score − 0.7, range − 3.0 to + 2.1, $P < 0.005$), and in integral bone of the lumbar spine (DXA: median BMD 1.141 g $cm^{-2}$, median *Z*-score − 0.6, range − 1.7 to + 1.3, $P < 0.0005$) and a significant reduction in integral bone of the femoral neck (DXA: median BMD 0.991 g $cm^{-2}$, median *Z*-score − 0.4, range − 2.1 to + 1.3, $P < 0.05$). Results of BMD measuurements are shown in Table I and illustrated as individual *Z*-scores in Figure 1. There was no significant correlation between time elapsed since completion of chemotherapy and *Z*-score at any site.

There was no significant change in serum testosterone, SHBG, LH and FSH with time from completion of chemotherapy in the 22 men who had these measurements performed on more than one occasion, so the mean of these measurements was used in these men, with the single measurements used in the remaining seven men. Serum testosterone was 15.2 ± 1.1 (range 8.2–35.4) nmol $l^{-1}$ (normal range 10–30 nmol $l^{-1}$), serum SHBG 38.2 ± 2.1 (range 16.0–70.5) nmol $l^{-1}$ (normal range 10–50 nmol $l^{-1}$), serum LH 9.7 ± 0.7 (range 5.0–19.0) IU $l^{-1}$ (normal range 2.0–10.0 IU $l^{-1}$) and serum FSH 18.1 ± 1.6 IU $l^{-1}$ (range 6.5–39.5) IU $l^{-1}$ (normal range 1.0–5.0 IU $l^{-1}$). The results of serum testosterone, SHBG, LH and FSH are illustrated in Figure 2. Results were also expressed as serum testosterone/SHBG ratio (0.4 ± 0.0, range 0.2–0.7).

There was a significant positive correlation between serum testosterone and *Z*-score of lumbar spine BMD ($r = 0.37$, $P = 0.02$) and of femoral neck BMD ($r = 0.41$, $P < 0.02$). There was a significant negative correlation between serum testosterone and *Z*-score of forearm BMC ($r = -0.45$, $P < 0.01$) but a non-significant positive correlation between serum testosterone and *Z*-score of forearm BMC/LBM ($r = 0.21$, $P = 0.14$). There was no significant correlation between *Z*-score at any site and serum SHBG, LH or FSH. There was a significant positive correlation between serum testosterone/SHBG ratio and *Z*-score of femoral neck BMD ($r = 0.44$, $P < 0.01$) and a significant negative correlation between serum testosterone/SHBG ratio and *Z*-score of forearm BMC ($r = -0.31$, $P < 0.05$). There was no significant correlation between this ratio and *Z*-score of lumbar spine BMD or of forearm BMC/LBM.

There was no significant difference in *Z*-score at any site between those men who had received MVPP ($n = 12$) and those who had received hybrid ($n = 17$) chemotherapy.

There was no significant difference in *Z*-score at any site between those men who had received eight cycles ($n = 20$) and those who had received 5–7 cycles ($n = 9$) of chemotherapy.

### Discussion

We have demonstrated a significant reduction in BMD in men who have previously received chemotherapy for Hodgkin's disease. The reduction in BMD affects both cortical bone, measured as the forearm by SPA, and integral bone, measured at lumbar spine and femoral neck by DXA. Measurements of BMD performed in men from our local population have been confirmed to correspond well with published reference data, indicating that expressing the BMD measurements as *Z*-scores derived from these published reference data is appropriate. We therefore feel that the reduced BMD demonstrated at the three sites is a true observation, although it is not in accordance with the findings of Kreuser *et al.* (1992), who reported that men who had

**Table I** Bone mineral density measurements and *Z* scores

| *Patient* | *SPA (g cm⁻¹)* | *SPA Z-score* | *SPA/LBM (g cm⁻¹ kg⁻¹)* | *SPA/LBM Z-score* | *DXA L2–L4 (g cm⁻²)* | *DXA L2–L4 Z-score* | *DXA femoral neck (g cm⁻²)* | *DXA femoral neck Z-score* |
|---|---|---|---|---|---|---|---|---|
| 1 | 1.680 | − 1.5 | 0.900 | − 0.8 | 1.163 | − 0.5 | 1.125 | 0.2 |
| 2 | 1.941 | 0.2 | 0.910 | − 0.9 | 1.049 | − 1.4 | 0.789 | − 2.1 |
| 3 | 1.595 | − 1.7 | 0.888 | − 1.0 | 0.921 | − 1.7 | 1.088 | 0.3 |
| 4 | 1.885 | 0.0 | 0.944 | − 0.6 | 1.145 | − 0.8 | 0.901 | − 1.2 |
| 5 | 2.134 | 0.4 | 1.043 | 0.4 | 1.466 | 1.3 | 1.105 | 0.0 |
| 6 | 1.585 | − 1.7 | 0.916 | − 0.7 | 1.157 | − 0.2 | 0.985 | − 0.4 |
| 7 | 1.648 | − 1.4 | 0.835 | − 1.4 | 1.281 | 0.2 | 1.129 | 0.5 |
| 8 | 1.641 | − 1.4 | 1.014 | 0.1 | 1.108 | − 0.3 | 0.828 | − 1.5 |
| 9 | 1.759 | − 0.8 | 0.919 | − 0.7 | 1.217 | − 0.2 | 0.942 | − 1.0 |
| 10 | 1.582 | − 1.3 | 0.831 | − 1.5 | 1.139 | − 0.5 | 0.936 | − 0.4 |
| 11 | 1.727 | − 0.1 | 0.659 | − 3.0 | 1.215 | − 0.8 | 0.847 | − 1.5 |
| 12 | 1.750 | − 0.8 | 0.756 | − 2.1 | 1.148 | − 1.3 | 1.061 | − 0.6 |
| 13 | 1.816 | − 0.6 | 0.990 | − 0.1 | 1.103 | − 0.6 | 0.869 | − 1.4 |
| 14 | 1.720 | − 0.8 | 0.974 | − 0.3 | 1.044 | − 1.0 | 1.029 | 0.3 |
| 15 | 1.661 | − 0.8 | 0.900 | − 0.9 | 1.234 | 0.0 | 0.881 | − 1.1 |
| 16 | 1.601 | − 1.8 | 1.257 | 2.1 | 1.046 | − 0.9 | 1.050 | − 0.2 |
| 17 | 1.664 | − 0.8 | 0.912 | − 0.8 | 1.036 | − 1.1 | 0.892 | − 1.0 |
| 18 | 1.802 | − 0.9 | 0.971 | − 0.3 | 1.327 | 0.7 | 1.183 | 0.9 |
| 19 | 1.799 | − 0.3 | 0.758 | − 2.1 | 1.231 | − 0.8 | 0.991 | − 0.7 |
| 20 | 2.184 | 0.8 | 1.180 | 1.5 | 1.146 | − 0.5 | 1.223 | 1.3 |
| 21 | 2.240 | 1.3 | 1.077 | 0.5 | 1.141 | − 0.8 | 0.991 | − 0.5 |
| 22 | 1.602 | − 0.6 | 0.826 | − 1.6 | 1.085 | − 0.8 | 0.927 | − 0.3 |
| 23 | 1.862 | − 0.2 | 0.939 | − 0.6 | 1.139 | − 0.7 | 1.078 | 0.2 |
| 24 | 1.727 | − 0.7 | 0.930 | − 0.7 | 1.044 | − 1.2 | 1.053 | 0.1 |
| 25 | 1.628 | − 0.8 | 0.788 | − 1.9 | 1.137 | − 0.8 | 0.832 | − 1.6 |
| 26 | 1.861 | − 0.4 | 0.906 | − 0.8 | 1.150 | − 0.6 | 0.908 | − 1.3 |
| 27 | 1.849 | − 0.8 | 0.974 | − 0.2 | 1.131 | − 0.6 | 1.110 | 0.2 |
| 28 | 1.480 | − 1.7 | 0.830 | − 1.5 | 1.274 | 0.4 | 1.229 | 1.6 |
| 29 | 1.828 | − 0.8 | 1.083 | 0.7 | 1.081 | − 0.6 | 0.931 | − 1.0 |

SPA, single photon absorptiometry; LBM, lean body mass; DXA, dual energy X-ray absorptiometry.

**Figure 1** BMD measurements illustrated as individual *Z*-scores.

**Figure 2** Serum testosterone, SHBG, LH and FSH.

received similar therapy for treatment of Hodgkin's disease showed no evidence of osteopenia.

The significant positive correlation between serum testosterone and *Z*-score at lumbar spine and femoral neck suggests that a reduction in serum testosterone, which may be a consequence of chemotherapy-induced Leydig cell damage, is associated with a reduction in integral BMD. Serum LH was elevated in 13 of the 29 (45%) men studied, in agreement with previous documentation of raised serum LH levels following chemotherapy for treatment of Hodgkin's disease (Whitehead *et al.*, 1982; Tsatsoulis *et al.*, 1987), indicating that Leydig cell function had been compromised in at least some of the men studied. The well-established association of hypogonadism and reduced BMD would support the possibility that subtle impairment of Leydig cell function, and hence a possible degree of mild hypogonadism, could result in a reduction of BMD. However, this mechanism does not explain the significant reduction in forearm cortical BMC demonstrated in the men studied as there is a significant negative correlation between serum testosterone and Z-score at this site. It is possible that integral and cortical bone may respond differently to hypogonadism of varying severity, or to alternative adverse factors, as demonstrated by the significant reduction in forearm cortical BMC, but lack of reduction in lumbar spine or femoral neck integral BMD, in women who had retained normal menstrual function following treatment of Hodgkin's disease (Redman *et al.*, 1988). In these women the reduction in cortical bone mass appears to be independent of chemotherapy-induced impairment of ovarian function and hence hypogonadism. The reduction of integral BMD in addition to cortical BMC in women with premature ovarian failure following treatment of Hodgkin's disease (Redman *et al.*, 1988) suggests that the reduced integral BMD is due to hypogonadism. However, the possibility that cortical and integral bone respond differently to hypogonadism is not in agreement with the finding that both cortical and integral BMD are reduced in men with severe hypogonadism due to idiopathic hypogonadotrophic hypogonadism (Finkelstein *et al.*, 1987) or hyperprolactinaemia (Greenspan *et al.*, 1986). Furthermore, if the osteopenia that we have demonstrated was due to hypogonadism, it might be expected that BMD would be further reduced with increasing time elapsed since completion of chemotherapy, and hence a longer duration of hypogonadism, but this is not the case in the men studied.

BMC, measured by SPA, provides a measure of bone mineral per unit length of forearm bones only, and takes no account of the width or depth of the bone; BMC/LBM can be used to provide an effective correction for both height and weight (Thomsen *et al.*, 1986). The significant negative correlation between serum testosterone and Z-score of forearm cortical BMC, in contrast to the significant positive correlation between serum testosterone and Z-score of lumbar spine and femoral neck integral BMD, may be due to a confounding effect of the size of the subject on the forearm cortical BMC. This is supported by the finding of a positive (but non-significant) correlation between serum testosterone and forearm cortical BMC when corrected for LBM, in accordance with the direction of the correlation between serum testosterone and Z-score at lumbar spine and femoral neck. This change in direction of the correlation from negative to positive when BMC is corrected for LBM may be due to the rather surprising and unexplained significant negative correlation between serum testosterone and LBM ($r = -0.36$, $P < 0.05$).

Short-term treatment of rats with methotrexate and doxorubicin has been shown to cause a significant reduction in vertebral trabecular bone volume and in bone formation rate (Friedlaender *et al.*, 1984), raising the possibility that chemotherapeutic agents may have a direct effect on bone turnover, and hence BMD, in man. Furthermore, glucocorticoid treatment can cause osteopenia (Baylink, 1983), and the steroid component of the chemotherapeutic regimens could be considered as a possible cause of osteopenia.

In conclusion, we have demonstrated that men who have received chemotherapy for Hodgkin's disease have reduced BMD. Possible causes include mild hypogonadism secondary to impaired Leydig cell function following chemotherapy, a direct effect of chemotherapy on bone, an effect of administration of high-dose glucocorticoid, or an effect of Hodgkin's disease *per se*. A reduction in femoral neck BMD by one standard deviation from the age-adjusted mean has been shown to increase the risk of hip fracture by a factor of 2.6 in women over the age of 65 years (Cummings *et al.*, 1993). Our findings demonstrate that men who have received chemotherapy for Hodgkin's disease may be at increased risk of bone fracture in later life, irrespective of the aetiology of the osteopenia.

We would like to thank Ric Swindell of the Medical Statistics Department, Christie Hospital NHS Trust, for performing the statistical analyses, and the North Western Regional Health Authority Research Grants Committee for providing generous financial support for bone densitometry.

### References

ADAMS, J.E., WHITEHOUSE, R.W., SILMAN, A., ADAMS, P.H. & KAY, C. (1992). Bone mineral density reference ranges across populations. In *Current Research in Osteoporosis and Bone Mineral Measurement*. Vol. II, Ring, E.F.J. (ed.) p. 14. British Institute of Radiology: London.

BAYLINK, D.J. (1983). Glucocorticoid-induced osteoporosis. *N. Engl. J. Med.*, **309**, 306–308.

BODDY, K., KING, P.C., HUME, R. & WEYERS, E. (1972). The relation of total body potassium to height, weight, and age in normal adults. *J. Clin. Pathol.*, **25**, 512–517.

CULLUM, I.D., ELL, P.J. & RYDER, J.P. (1989). X-ray dual-photon absorptiometry: a new method for the measurement of bone density. *Br. J. Radiol.*, **62**, 587–592.

CUMMINGS, S.R., BLACK, D.M., NEVITT, M.C., BROWNER, W., CAULEY, J., ENSRUD, K,. GENANT, H.K., PALERMO, L., SCOTT, J. & VOGT, T.M. (1993). Bone density at various sites for prediction of hip fractures. *Lancet*, **341**, 72–75.

FINKELSTEIN, J.S., KLIBANSKI, A., NEER, R.M., GREENSPAN, S.L., ROSENTHAL, D.I. & CROWLEY, W.F. (1987). Osteoporosis in men with idiopathic hypogonadotropic hypogonadism. *Ann. Intern. Med.*, **106**, 354–361.

FINKELSTEIN, J.S., KLIBANSKI, A., NEER, R.M., DOPPELT, S.H., ROSENTHAL, D.I., SEGRE, G.V. & CROWLEY, W.F. (1989). Increases in bone density during treatment of men with idiopathic hypogonadotropic hypogonadism. *J. Clin. Endocrinol. Metab.*, **69**, 776–783.

FINKELSTEIN, J.S., NEER, R.M., BILLER, B.K., CRAWFORD, J.D. & KLIBANSKI, A. (1992). Osteopenia in men with a history of delayed puberty. *N. Engl. J. Med.*, **326**, 600–604.

FRIEDLAENDER, G.E., TROSS, R.B., DOGANIS, A.C., KIRKWOOD, J.M. & BARON, R. (1984). Effects of chemotherapeutic agents on bone. 1. Short-term methotrexate and doxorubicin (adriamycin) treatment in a rat model. *J. Bone Joint Surg.*, **66-A**, 602–607.

GOTFREDSEN, A., JENSEN, J., BORG, J. & CHRISTIANSEN, C. (1986). Measurement of lean body mass and total body fat using dual photon absorptiometry. *Metabolism*, **35**, 88–93.

GREENSPAN, S.L., NEER, R.M., RIDGWAY, E.C. & KLIBANSKI, A. (1986). Osteoporosis in men with hyperprolactinaemic hypogonadism. *Ann. Intern. Med.*, **104**, 777–782.

HOROWITZ, M., WISHART, J.M., O'LOUGHLIN, P.D., MORRIS, H.A., NEED, A.G. & NORDIN, B.E.C. (1992). Osteoporosis and Klinefelter's syndrome. *Clin. Endocrinol.*, **36**, 113–118.

KREUSER, E.D., FELSENBERG, D., BEHLES, C., SEIBT-JUNG, H., MIELCAREK, M., DIEHL, V., DAHMEN, E. & THIEL, E. (1992). Long-term gonadal dysfunction and its impact on bone mineralization in patients following COPP/ABVD chemotherapy for Hodgkin's disease. *Ann. Oncol.*, 3 (Suppl. 4), 105–110.

LASKEY, M.A., CRISP, A.J., COLE, T.J. & COMPSTON, J.E. (1992). Comparison of the effect of different reference data on Lunar DPX and Hologic QDR-1000 dual-energy X-ray absorptiometers. *Br. J. Radiol.*, **65**, 1124–1129.

PARFITT, A.M. (1990). Interpretation of bone densitometry measurements: disadvantages of a percentage scale and a discussion of some alternatives. *J. Bone Min. Res.*, **5**, 537–540.

REDMAN, J.R., BAJORUNAS, D.R., WONG, G., MCDERMOTT, K., GNECCO, C., SCHNEIDER, R., LACHER, M.J. & LANE, J.M. (1988). Bone mineralization in women following successful treatment of Hodgkin's disease. *Am. J. Med.*, **85**, 65–72.

STEPAN, J.J., LACHMAN, M., ZVERINA, J., PACOVSKY, V. & BAYLINK, D.J. (1989). Castrated men exhibit bone loss: effect of calcitonin treatment on biochemical indices of bone remodelling. *J. Clin. Endocrinol. Metab.*, **69**, 523–527.

THOMSEN, K., GOTFREDSEN, A. & CHRISTIANSEN, C. (1986). Is postmenopausal bone loss an age-related phenomenon? *Calcif. Tissue Int.*, **39**, 123–127.

TSATSOULIS, A., WHITEHEAD, E., ST. JOHN, J., SHALET, S.M. & ROBERTSON, W.R. (1987). The pituitary-Leydig cell axis in men with severe damage to the germinal epithelium. *Clin. Endocrinol.*, **27**, 683–689.

WAHNER, H.W., RIGGS, B.L. & BEABENT, J.W. (1977). Diagnosis of osteoporosis: usefulness of photon absorptiometry of the radius. *J. Nucl. Med.*, **18**, 432–437.

WHITEHEAD, E., SHALET, S.M., BLACKLEDGE, G., TODD, I., CROWTHER, D. & BEARDWELL, C.G. (1982). The effects of Hodgkin's disease and combination chemotherapy on gonadal function in the adult male. *Cancer*, **49**, 418–422.